# Genetic Permissiveness and Dietary Glycemic Load Interact to Predict Type-II Diabetes in the Nile rat (*Arvicanthis niloticus*)

**DOI:** 10.3390/nu11071538

**Published:** 2019-07-06

**Authors:** Avinaash Subramaniam, Michelle Landstrom, K. C. Hayes

**Affiliations:** Biology Department, Brandeis University, Waltham, MA 02453, USA

**Keywords:** type 2 diabetes, Nile rat, animal models, epidemiology, quintiles, nutrition, glycemic load

## Abstract

**Objective**: The Nile rat (Arvicanthis niloticus) is a superior model for Type-II Diabetes Mellitus (T2DM) induced by diets with a high glycemic index (GI) and glycemic load (GLoad). To better define the age and gender attributes of diabetes in early stages of progression, weanling rats were fed a high carbohydrate (hiCHO) diet for between 2 to 10 weeks. **Methods**: Data from four experiments compared two diabetogenic semipurified diets (Diet 133 (60:20:20, as % energy from CHO, fat, protein with a high glycemic load (GLoad) of 224 per 2000 kcal) versus Diets 73 MBS or 73 MB (70:10:20 with or without sucrose and higher GLoads of 259 or 295, respectively). An epidemiological technique was used to stratify the diabetes into quintiles of blood glucose (Q1 to Q5), after 2–10 weeks of dietary induction in 654 rats. The related metagenetic physiological growth and metabolic outcomes were related to the degree of diabetes based on fasting blood glucose (FBG), random blood glucose (RBG), and oral glucose tolerance test (OGTT) at 30 min and 60 min. **Results**: Experiment 1 (Diet 73MBS) demonstrated that the diabetes begins aggressively in weanlings during the first 2 weeks of a hiCHO challenge, linking genetic permissiveness to diabetes susceptibility or resistance from an early age. In Experiment 2, ninety male Nile rats fed Diet 133 (60:20:20) for 10 weeks identified two quintiles of resistant rats (Q1,Q2) that lowered their RBG between 6 weeks and 10 weeks on diet, whereas Q3–Q5 became progressively more diabetic, suggesting an ongoing struggle for control over glucose metabolism, which either stabilized or not, depending on genetic permissiveness. Experiment 3 (32 males fed 70:10:20) and Experiment 4 (30 females fed 60:20:20) lasted 8 weeks and 3 weeks respectively, for gender and time comparisons. The most telling link between a quintile rank and diabetes risk was telegraphed by energy intake (kcal/day) that established the cumulative GLoad per rat for the entire trial, which was apparent from the first week of feeding. This genetic permissiveness associated with hyperphagia across quintiles was maintained throughout the study and was mirrored in body weight gain without appreciable differences in feed efficiency. This suggests that appetite and greater growth rate linked to a fiber-free high GLoad diet were the dominant factors driving the diabetes. Male rats fed the highest GLoad diet (Diet 73MB 70:10:20, GLoad 295 per 2000 kcal for 8 weeks in Experiment 3], ate more calories and developed diabetes even more aggressively, again emphasizing the Cumulative GLoad as a primary stressor for expressing the genetic permissiveness underlying the diabetes. **Conclusion**: Thus, the Nile rat model, unlike other rodents but similar to humans, represents a superior model for high GLoad, low-fiber diets that induce diabetes from an early age in a manner similar to the dietary paradigm underlying T2DM in humans, most likely originating in childhood.

## 1. Introduction

Type-2 Diabetes Mellitus (T2DM) is a pernicious metabolic disease whose prevalence has quadrupled over the past 35 years and is still growing at an alarming pace worldwide. The disease is predicted to affect 438 million people by the year 2030, becoming the seventh leading cause of death, with roughly 80% of these deaths occurring in low and middle-income countries [1,2,3,4,5,6]. 

In order to study the incidence of diseases like T2DM, epidemiological studies are often conducted in large human populations. These extensive data sets apply meta-analyses on different parameters and biomarkers to offer insight for potential nutritional interventions that might be used to prevent or treat the disease. Such massive data sets allow stratification into quantiles (e.g., tertiles, quintiles, deciles) to suggest specific gene and/or diet x gene interactions affecting disease across the entire population under study. However, such studies are observational and are often poorly controlled in terms of dietary history. Additionally, in observational studies, it is not possible to control how subjects are assigned to subgroups or the specifics of diet history related to each group [7,8,9]. Systematic errors can also occur, relating to omission of foods consumed by individuals, errors in estimating portion sizes and over-or-under reporting because of social constraints [8,9,10,11]. Dietary variables are rarely dichotomous; often, but not always, the entire population is exposed to some degree to any given nutrient. Diet is also varied over time, making diet x gene interactions problematic [9] and the food frequency questionnaire is less reliable than feeding a controlled diet throughout an entire study.

To avoid problems associated with design, the Nile rat has been identified as a unique model of T2DM that rapidly develops all the requisite aspects of the disease when consuming a hiCHO, high GLoad, low-fiber diet in a manner similar to that of the human experience [12,13,14], and unlike most rodent models that require a high-fat diet to induce diabetes by way of obesity [10,11,15,16,17,18,19,20,21,22,23,24,25,26,27,28,29,30,31]. In many conventional preclinical nutritional studies, standard laboratory chow is fed to the animal model. However, chow is of limited value from a nutritional perspective because macronutrient composition is not clearly defined [13,14,32,33]. The manipulation of individual CHO, fats, or proteins cannot be realized, and interactive effects of nutrients cannot be studied for their impact on diabetes induction. Hu [34] acknowledges that the high level of intercorrelation between nutrients makes it difficult to examine their separate effects and suggest using dietary pattern analyses. However, the differences in nutrients between dietary patterns makes the approach unspecific as to the particular nutrients responsible for the observed differences in disease risk [34]. Semipurified diets are more exacting in nutritional studies because the qualitative and quantitative aspects and the macronutrient and micronutrient consumption can be controlled [32,33]. A hiCHO semipurified diet with a high GLoad fed to Nile rats is more diabetogenic than rat chow and, hence, diabetes develops more quickly. Insight into the Nile rat glucose response to diet can be further determined by manipulating the % energy ratios of macronutrients (CHO:Fat:Protein), fiber, and polyphenol content, as each of these are controlled and explored precisely for their impact on induction or protection against T2DM [12,14,35]. 

Studies with humans have also consistently shown that CHO and the GLoad are strongly related to T2DM and the Metabolic Syndrome (MetS) [10,11,36,37,38,39,40,41,42,43]. In fact, restricting CHO as opposed to restricting fat produces the greatest reduction in blood glucose, be it postprandially or with other measures of glucose overload, including HbA1c. Refined CHO, in particular, can produce more harmful metabolic effects than do equivalent calories from saturated fats [10,11,13,30,38,44,45,46,47,48,49,50,51,52,53,54]. Such is the case in Nile rats. 

Accordingly, the present study was designed to utilize an appropriate CHO model, where a controlled hiCHO diet could be utilized to model CHO-induced diabetes, to explore the inherent genetic attributes towards individual responsiveness to MetS and diabetes induction. The focus was not on mechanisms so much as on establishing the best way to detect gene-diet influences on the earliest onset and progression of to assure the enhanced design of future studies.

## 2. Materials and Methods 

### 2.1. Animal Model (Nile rat)

The Nile rat is a preferred animal model for diet-induced T2DM in humans for several reasons. Firstly, this rodent is highly adaptable to a captive environment, where it breeds well even as it develops metabolic disease. This situation mimics the increasingly sedentary environment of humans today. In the wild, the Nile rat lives in the dry savanna and grasslands and feeds mainly on grasses, leaves and stems of flowering plants and arthropods. In addition, the Nile rat has ample exercise in wild. Due to food being a limited resource and increased exercise (as a consequence of avoiding predation and searching for food), the Nile rat in the wild does not develop diabetes when consuming a fiber-rich, low GLoad diet based on native grasses and bushes in its semi-arid desert environment [55,56,57,58]. 

However, in captivity with abundant food and no predators, even the Nile rat fed rat chow slowly develops MetS that eventually evolves into T2DM with all the pertinent features of the human condition: insulin resistance, hyperinsulinemia, hypertension linked ultimately to kidney failure, elevated TG with decreased HDL, and eventually hyperglycemia and beta cell failure resulting in depressed insulin and end-stage diabetes that includes severe ketosis [12,13,14,59,60], peripheral polyneuropathy [61], and cardiovascular disease [60,62]. They are also useful in studying diabetic lesions of the eye, including cataracts and retinopathy [63,64,65,66] and eventual renal failure as a consequence of advanced diabetes [13,67]. 

### 2.2. Semipurified diets

In order to overcome a major criticism of poorly-defined chow diets and observational epidemiological studies in humans, the Nile rats were fed well-defined semipurified diabetogenic diets previously used to document the individual variation associated with *genetic permissiveness* that is evoked when a cohort of these rats consumes the same high GLoad diet [12,13,14]. The GI and GLoad were defined as previously described [14], using units derived for CHOs fed to humans with dietary GLoads adjusted per 2000 calories of Nile rat diets. Measuring daily food intake provided necessary details of energy intake that were expressed as metabolic characteristics related to clinical outcomes, including total calories consumed, body weight gain, RBG and FBG as well as OGTT, and organ pathology at necropsy after 8 weeks or 10 weeks on diet, to better express genetic differences underlying the disease process. Table 1 details the diet compositions used in all experiments.

### 2.3. Procedures 

#### 2.3.1. Experiment 1: Early Diabetes Onset in both Male and Female Rats Fed Diet 73MBS (70:10:20, CHO:Fat:Protein %energy) for 2 Weeks or 4 Weeks 

The initial experiment identified Nile rats as *resistant* and *susceptible* phenotypes at an early age by stratifying the diabetes into quintiles based on the 30-min oral glucose tolerance test (OGTT). Young male and female pups were fed a hiCHO diet (Diet 73MBS, 70:10:20 GLoad 259/2000 kcal), and their diabetes assessment included an OGTT in order to examine diabetes severity beyond the RBG and FBG profiles. All together 255 male and 247 female pups were weaned at 3 weeks old (30–32 g body weight), but uniquely in this study littermates were housed together for the 2 weeks or 4 weeks and fed Diet 73MBS (70:10:20 containing sucrose). This diet represents one of three CHO loads tested throughout the four experiments, representing the diet with an intermediate GLoad (259/2000 kcal due to the lower GI of sucrose vs dextrose or starch) all in the absence of dietary fiber. One cohort of rats (165 males and 138 females) was fed the diet for 2 weeks (until 5 weeks old), at the end of which they were subjected to OGTT (225 μL/100 g rat with a solution containing 10.6 g dextrose dissolved in 6 mL water, representing 1.9 g dextrose/ kg body weight). A second cohort of weanlings (90 males, 109 females) was continued on the hiCHO diet for 4 weeks (until 7 weeks old) before being subjected to an OGTT. 

#### 2.3.2. Experiment 2: Diabetes Progression Tracked 90 Male Nile Rats for 10 Weeks into Sexual Maturity while Fed Diet 133 (60:20:20, CHO:Fat:Protein %Energy)

Once subgroups of *resistant* and *susceptible* rats were identified as early as 2 weeks and 4 weeks in Experiment 1, longer induction periods were evaluated to allow the diabetes to develop more fully in males, which are more sensitive than females to the diabetes [59,66]. 

Accordingly, Experiment 2 examined 90 male pups that were separated at 3 weeks old (average: 30–32 g body weight) and fed Diet 133 for 10 weeks (until 13 weeks old). Data were pooled from 7 different studies using only the control rats fed the 60:20:20 diabetogenic Diet 133. After 10 weeks these 90 rats were necropsied and their data stratified by quintiles of their RBG, previously identified as the best index of diabetes progression [12,14] and utilized by others as well [66].

Other standard measures of nutritional status were collected, including body weight gain, daily food and water intakes and food efficiency. In addition, three blood glucose parameters were compared, i.e., the RBG, OGTT including the FBG at zero time and at 30 min after 6 weeks, as well as FBG at zero time, 30 min, 60 min after 10 weeks of diet challenge, followed by necropsy in week 10. Total cholesterol (TC) and triglycerides (TG) were measured from plasma separated at necropsy. RBG was stratified by quintiles from low to high T2DM (*n =* 18 per RBG subgroup), in order to rank the diabetes according to rat *genetic permissiveness* across the subgroups. Data was shown in Table 2. 

#### 2.3.3. Experiment 3: Diabetes Progression Followed 8 Weeks in 32 Male Nile Rats Fed Diet 73MB/73MBS (70:10:20, CHO:Fat:Protein %Energy) at Increased GLoad

After completion of the 10-week study with Diet 133 (60:20:20, GLoad 224, Experiment 2) a shorter feeding trial of 8 weeks was tested in males to determine how a shorter period at higher GLoad (70:10:20) would compare for rate of diabetes induction. Thus, 32 male pups (selected as controls from 3 ongoing studies) also were weaned at 3 weeks (30–32 g body weight) and were fed similar semipurified diets (70:10:20), but with higher GLoads (Diet 73MB with dextrose for 2 groups, GLoad 295, or 73MBS with sucrose for 1 group, GLoad 259). Both diets were moderately calorically dense, 3.98 kcal/g dry diet. Standard measures were instituted, including body weight gain, daily food and water intakes and food efficiency. In addition, blood glucose parameters included RBG after 6 and 10 weeks on diet, with an OGTT that included FBG at zero time and 30 min after 4 weeks, as well as an OGTT (FBG at zero time and after 30 min and 60 min) after 8 weeks, followed by necropsy. TC and TG were measured from plasma collected at necropsy.

#### 2.3.4. Experiment 4: Compared Diabetes in 30 Female Nile Rats after 3 Weeks while Fed Diet 133

In a fourth short-term experiment, females were fed Diet 133 (60:20:20) with its lower GLoad to compare with those fed 73MBS in Experiment 1 as well as against males fed Diet 133 for a longer term in Experiment 2. Thus, 30 female pups were separated at 3 weeks old (30–32 g body weight). All rats were fed the same Diet 133 used in Experiment 2. Blood glucose parameters RBG at 3 weeks on diet, and an OGTT with FBG at zero-time and 30 min during week 3 (6 weeks old), when they were necropsied. 

All experiments and procedures were approved by the Brandeis University Institutional Animal Care and Use Committee (IACUC). 

## 3. Results

### 3.1. Experiment 1: Diabetes Detected Early between 5–7 Weeks Old with Diet 73MBS (70:10:20)

The initial experiment described early diabetes onset and progression in both male and female Nile rats between 5 and 7 weeks of age in 502 rats. The diabetes began aggressively in the first two weeks with this hiCHO diet challenge (73MBS 70:10:20, GLoad 259/2000 kcal). Stratification by quintiles of the 30-min OGTT identified *genetic permissiveness* as a key aspect of susceptibility expressed at an exceptionally young age. 

Data in Table 3 represents males and females, respectively, and highlight the relative burden of glucose loading and/or insulin resistance (IR) that begins early in life for the Nile rat fed a hiCHO challenge diet with a high GLoad, in this case 259/2000 kcal of diet compared to approximately 125/2000 kcal for an average North American diet. On the one hand, at both 2 weeks and 4 weeks, a 5 g increment in body weight between Q1 to Q5 in males was associated with a 3- to 4-fold increase in 30-min OGTT, suggesting that the post-weaning IR risk develops early in the model associated with greater weight gain. By contrast, and something of a paradox, consumption of hiCHO between 2 to 4 weeks in these post-weaning males led to a 10 g rise in body weight (60 g to 70 g) across all quintiles, but the relative OGTT and, to lesser extent FBG, in all quintiles except Q5 *improved (decreased)*, especially within the two lowest ‘*resistant*’ quintiles (Q1,Q2) of diabetes. This suggests that early on in the hiCHO challenge, 80% of the male rats appeared to improve their glucose tolerance [i.e., they were *resisting* diabetes induction] as they aged during this 2-week window of early adaptation to the hiCHO diet. Thus, even though all pups consumed the same diet and began the study at the same weight in each quintile, most actually were becoming less diabetic initially after 2 to 4 weeks. Females, by contrast, revealed less than half the weight gain observed in males (about 3 to 4 g) during that 4-week timeframe, but they followed the same general response seen in males, because all quintiles improved their 30-min OGTT curve between 2 and 4 weeks. 

Furthermore, after 2 weeks of diet challenge (5 weeks old), about 60% of both males and females revealed a ‘normal’ FBG at <60 mg/dl at the start of the OGTT following overnight food restriction. After 4 weeks of diet (7 weeks old), 80% of males still had normal FBG <60 mg/dl, but surprisingly only 40% (Q1 and Q2) of the females still displayed FBG <60 mg/dl. Nonetheless, like males the 30-min OGTT and FBG for most females also tended to be lower after 4 weeks than after 2 weeks on diet so that both genders ironically tended to become *less* acutely diabetic during the early stages of the hiCHO challenge diet introduced at weaning as measured by the FBG and OGTT indices. This might suggest that improvement in liver efficiency for glucose processing (declining FBG), and or the peripheral capacity to utilize glucose (by expanding muscle mass) were enhancing insulin sensitivity (IS). The greatest expression of the glucose overload and IR associated with diabetes (based on increased FBG and RBG) occurred immediately after weaning during this 4-week hiCHO challenge period independent of gender, then stabilized as growth rate slowed between 5–7 weeks of age (Table 3**)**. Ironically, the growth data for females may indicate that they fail to improve their FBG because they do not grow muscle, adipose or liver tissues (body mass) fast enough to dispose of the diet glucose load, but further studies will be needed to substantiate this hypothesis.

### 3.2. Experiment 2. T2DM Allowed to Develop in Males to 13 Weeks of Age

As a follow-up to the limited period of study in young male and female rats in Experiment 1, the data from Experiment 2 emphasize several points based on diabetes induction for male Nile rats fed a hiCHO challenge for 10 weeks (Table 4). 

The data in Table 4 represent pooled responses for control rats all fed the same diabetogenic diet (Diet 133, 60:20:20) from 7 studies. *Genetic permissiveness* was illustrated by stratifying rats into quintiles of RBG, revealing a mean of 53mg/dl glucose for rats in Q1 to 439 mg/dl in Q5. As in Table 3 with younger rats, the diabetes in these older, continuously-challenged males continued to progress in accord with their rising RBG across quintiles (Q1 to Q5). Thus, diabetes in Q5 became modest to severe as early as 6 weeks into the study (Table 4). This consolidation and stratification of data by quintiles clearly illustrates the *genetic permissiveness* of the host metabolism towards diabetes noted previously [13,14] including Experiment 1 (70:10:20), where diabetes was based on FBG and 30-min OGTT. This second experiment demonstrates why RBG is a preferred index for diabetes assessment and why it is consistently a strong predictor of the diabetes observed at necropsy after 8–10 weeks of diet challenge in these studies [13,14,66].

The RBG in Experiment 2 (not available in Experiment1) increased to greater than 150 mg/dl and 425 mg/dl, respectively, in the two top quintiles (Q4 and Q5) of RBG, and was much more responsive than the FBG, and less cumbersome to assess than the OGTT. In addition, rats least prone to diabetes (Q1 and Q2, both with RBG less than 75 mg/dl), can be considered *permissively resistant* compared to Q3–Q5. Furthermore, the RBG in *resistant* rats actually appeared to regress slightly between 6 weeks and 10 weeks as the weight gain in these rats slowed, whereas *susceptible* rats in Q4 and Q5 revealed substantial increases in RBG as they continued their rapid growth rate in that 4 week span. This confirms the implication from Experiment 1 that *resistant* rats in Q1-Q2 were characterized by glucose metabolism that was adjusting successfully as they matured, even as metabolism became progressively impaired and eventually failed in Q4 and Q5 as they became *susceptible* to advanced diabetes. 

**Figure 1:**
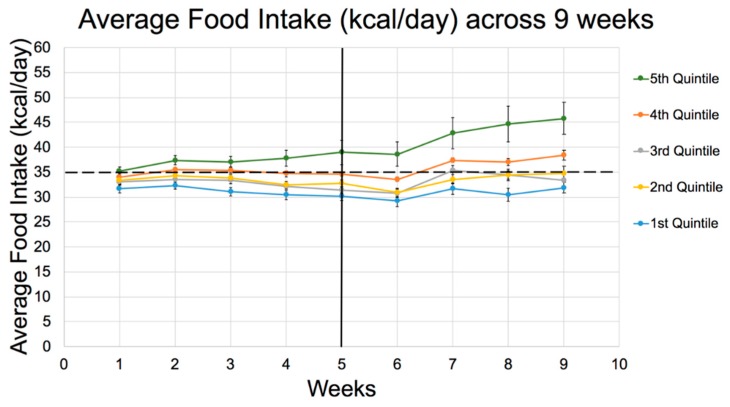
Food Intake (kcal/day) for 9 weeks on Diet 133 (60:20:20 CHO:fat:protein %energy) by quintiles in Experiment 2. Dotted line represents the average food intake threshold per day (35 kcal/day), exceeded only by Q4 and Q5. Q5 is also significantly greater than Q1 from the first week and all others including Q4 (*p* < 0.05) from week 6 onwards. The dip in all curves at 6 weeks reflects the stress as a result of OGTT that week. See Table 4 for detailed differences between quintiles from week 1.

Third, the body weight data from rats representing all 5 quintiles of susceptibility (Table 4), indicate that the rats destined to become *susceptible* (in Q4 and Q5) displayed more rapid weight gain from the outset and outgained the *resistant* rats at all intervals. This was true despite the fact that all pups were weaned at the same body weight (average: 30 g to 32 g) when Diet 133 (60:20:20 CHO:Fat:Protein %energy)was started. These growth data, in turn, parallel increases in food intake (Figure 1) that were already apparent after the first week (Table 4). 

Furthermore, the GLoad per rat/day increased progressively from Q1 to Q5 in accord with food intake (kcal/day). The Cumulative GLoad/rat, along with energy intake and weight gain, was one of the main predictors of an elevated RBG by the end of the study. However, note that GLoad (food intake) was not different between Q1–Q4 once adjusted for BMI, suggesting that body size itself influenced hunger and food consumption, possibly more than macronutrient composition. Only severely diabetic rats in Q5 still revealed abnormally elevated food and water consumption after adjusting for BMI (Table 4). 

Another point of interest is the fact that fasting blood glucose (FBG), observed at 0 min in the OGTT after overnight 16-h food restriction, was essentially normal in all the rats up to 6 weeks, and only began to show abnormal fasting glycemia in the most susceptible Q5 at 10 weeks. This was associated with increased liver weight (Table 4), consistent with our earlier studies [12,13,14], which suggests that FBG may reflect the degree of fatty liver [69]. Thus, non-alcoholic fatty liver disease (NAFLD) appeared to be associated with and signaled by the FBG because they increased in tandem. The normal range for fasting glucose in the Nile rat is between 40 and 60 mg/dl, when the livers are small and free of fat accumulation.

Q5 vs Q1 demonstrated a +45% difference in enlarged fatty liver, an equally enlarged kidney associated with excessive water consumption, and expanding total adipose pools of 10–20%, before declining in Q5 when rats succumbed to the catabolism of ketosis. By the same token, plasma lipids were severely elevated in Q5 with higher TC and TG associated with fatty liver, reflecting high insulin resistance linked to peak elevations in 30-min OGTT (i.e., an impaired OGTT clearance rate) [12,14]. It would appear that the Nile rat model offers an excellent opportunity to document the specific mechanisms involved with CHO-induced NAFLD progression/regression without modification beyond the CHO diet composition/challenge described here. 

The developing stages of diabetes in Experiment 2 were further defined by the oral glucose tolerance test (OGTT) conducted after 6 weeks and again after 10 weeks of diet challenge (Table 4). Considering the constraints of applying the OGTT, it was noted previously that the 30-min OGTT response is an earlier signal than RBG for detecting IR and the onset of diabetes but not as useful as RBG for predicting the diabetes documented at necropsy [13,14]. 

To that point, note that both after 6 weeks, and again at 10 weeks, the most *resistant* rats remained below 150 mg/dl at 30-min OGTT. This 30-min parameter increased steadily as susceptibility increased across quintiles with the most *susceptible* rats (Q5) having extremely elevated 30-min glucose in excess of 350 mg/dl. Also notice that the profiles of the 6 week OGTT for any given quintile generally was comparable to the 10 week OGTT, indicating that by 6 weeks the diabetes was at full force and did not become exacerbated during the following 4 weeks, at least in terms of the acute oral glucose challenge during a hiCHO consumption based on Diet 133 (60:20:20). However, the pathology encountered after 10 weeks has indicated that the changes in organs, including liver, kidney, adipose, and blood lipids become progressively worse between 6 weeks and 10 weeks (Unpublished Data and [13,14]). Comparing the 30 vs. 60-min glucose during OGTT recorded after 10 weeks, indicates that the 30-min response typically represents the peak in the OGTT curve for male rats in our typical 10-week assay.

Organ weights focused on those most affected by diabetes progression (Table 4). As demonstrated previously, the increased liver weight (NAFLD) was observed only in the most *susceptible* rats, associated with enlarged kidneys and cecum. Adipose tissue also reflected advancing diabetes, with perirenal and brown fat pads being the most dynamic fat depots. Both of these fat pads tend to increase modestly with an increasing dietary GLoad until advanced diabetes is encountered, as seen in Q5, where catabolic effects of ketosis are evident in association with accelerated lipolysis and reduced fat accumulation, with the exception of liver fat (size), which continues to enlarge. The total fat defined by the three fat pads, though more subtle, is a useful index to track the diabetes, as well. Carcass weight obtained once all the organs have been removed is a convenient index of the lean body mass in this case, indicating that the least diabetic rats have the greatest lean carcass mass as percent body weight. This index progressively declined across quintiles as the diabetes increased and catabolic activity of advanced diabetes consumed not only the adipose tissue, but also muscle mass. In terms of length, note that the smallest rats with the least food intake and body weight gain also had a slightly reduced body length (linear growth rate], whereas greater food intake and growth rate are reflected in greater length. The body fat and body length relationship is further captured by the body mass index, which revealed a progressive, modest increase across the first four quintiles, then declined in the most susceptible quintile (Q5) as ketosis developed and body tissues were catabolized (Table 4). Note that in no way can diabetic rats be considered ‘obese’ in the classical sense of an expanded BMI, with less than 8% difference between Q1 and Q4 rats, which scarcely differed from the normal *resistant* rats identified as Q1 (i.e., a range of roughly 20–24 kg/m^2^ BMI in human equivalents). This vastly differs from mice or conventional rats fed high-fat diets to induce their T2DM or the relationship of obesity to diabetes in susceptible humans, where BMI typically ranges from 35–50 kg/m^2^ in human equivalents.

Plasma lipid increases are in keeping with the other data on food intake, fatty liver development, and diabetes progression, as cholesterol and triglycerides became elevated only in Q4 and Q5 that were most susceptible to diabetes.

In summary, the full gamut of Type-2 Diabetes physiology is presented in the comparative profile of these rats under this dietary challenge (the 60:20:20 diet), allowing one the potential to intercede at any stage of the disease to explore either the cause of the diabetes *across the range of inherent genetic susceptibility,* or to intervene with drug therapy or attempt modification of the diabetes by diet.

### 3.3. Experiment 3: Diet 70:10:20 for 8 Weeks in Males 

Experiment 3 pooled 3 control groups of rats fed the highest CHO diet (73MB, 73MBS at 70:10:20) similar to that fed in Experiment 1. Two groups were fed 73MB and one 73MBS, but results did not differ between them. However, compared to Experiment 2 and its 60:20:20 composition, the diets provided a greater GI and GLoad, at 259 (73MBS, sucrose) or 295 (73MB, dextrose), but the study lasted only 8 weeks. In this truncated experiment, the diabetes developed even more aggressively, further suggesting the greater GLoad was a major contributor to expressing the inherent *genetic permissiveness* and diabetes outcome in this model. Data for these male rats in Experiment 3 indicate that *susceptibility* was shifted to the ‘left’ to appear in lower quintiles, specifically in Q3 and even slightly into Q2 (Table 5). 

By contrast, rats in the first 2 quintiles in Experiment 2 (60:20:20) remained *resistant,* while those in Q3 were prediabetic, and Q4 and Q5 were *susceptible* (Table 4). Hence, these lower-fat, higher-CHO diets (Diet 73MB, 73MBS) in Experiment 3 caused rats previously rated as *resistant* in the previous experiment to register as prediabetic, *indicating that increasing carbohydrate by replacing fat energy* increased the GLoad, reduced the fat calories, and provided less protection against T2DM that also developed earlier in the trial.

**Figure 2:**
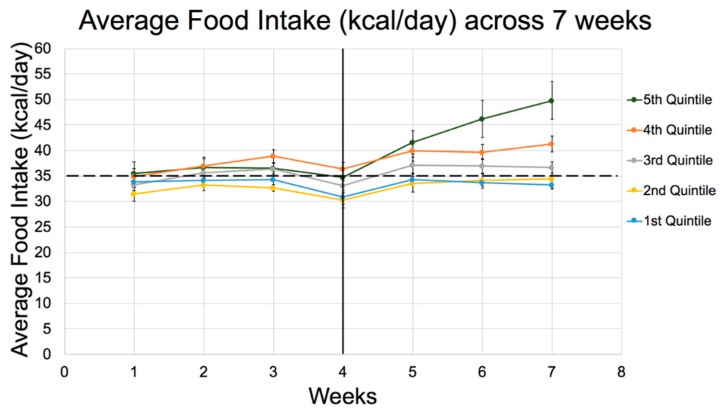
Average Food Intake (kcal/day) for 7weeks on Diet 73 (70:10:20, CHO:fat:protein % energy) by quintiles in Experiment 3. Dotted line represents the average food intake threshold per day (35 kcal/day), exceeded only by Q4 and Q5. The dip in food intake at 4 weeks reflects the stress as a result of OGTT conducted that week. See Table 5 for detailed differences between quintiles apparent from week 1.

In support of the above observations, the 30-min OGTT after 8 weeks was greater across quintiles for rats fed Diet 73MB (70:10:20, GLoad 295) (Table 5) compared to Diet 133 (60:20:20, GLoad 224) (Table 4), even though the OGTT was conducted 2 weeks earlier in Experiment 3 than Experiment 2, indicating the diabetes peaked much earlier in Experiment 3. Furthermore, whereas the trend in RBG at 10 weeks in Experiment 2 (60:20:20) tended to mirror the kcal/day intake across quintiles with only rats in Q4 and Q5 experiencing obvious hyperphagia early on, rats fed 70:10:20 revealed less extreme differences in hyperphagia between quintiles. However, all quintiles appeared to consume 10–20% more calories than the equivalent cohorts in Experiment 2, with excessive intake already evident in Q5 in the fifth week, e.g., compare energy intakes and food efficiency for weeks 1-6 between the two studies. In sum, the total average kcal/day for all quintiles was greater for 70:10:20 than in 60:20:20. Like Experiment 2, Q5 rats in Experiment 3 experienced an upsurge in food intake, this time after 4 weeks (Figure 2). 

The rats fed 70:10:20 also tended to have reduced feed efficiency, a characteristic of advancing diabetes (Table 5), i.e., gained similar weight but ate more calories to accomplish that weight gain when eating a higher percentage of CHO calories. In other words, higher percent energy from CHO raised kcal intake somewhat, but directed fewer calories towards weight gain, including fat storage, while resulting in more diabetogenic blood glucose parameters. Recall that rats in Experiment 3 (Table 5) were exposed to diet for 2 weeks less (terminated 2 weeks earlier) than those in Experiment 2 (Table 4), so liver size (fat), adipose depots, and terminal plasma lipids are likely under-represented by numbers reported for Experiment 3 when compared directly to rats in Experiment 2. 

This implies that a diet higher in CHO and extremely low in fat was more detrimental to metabolic controls, substantially affecting hunger and satiety for rats in Q5. This shift in macronutrient composition to greater CHO increased food intake and diabetes expression. Furthermore, because the hyperphagia exhibited by Q5 rats fed 70:10:20 was already prevalent after 5 weeks, the detrimental impact appeared 1 week earlier than that observed when rats consumed 60:20:20 with less CHO. Also, all Q3–Q5 rats fed 70:10:20 in Experiment 3 exhibited an average food intake which was above the 35 kcal/day threshold observed in Experiment 2 (60:20:20). Accordingly, the lower fat (higher CHO) diet increased appetite, which would support the notion that fat helps to suppress appetite and caloric intake [48], at least in the appropriate model. 

### 3.4. Experiment 4: Females Fed 60:20:20

Table 6 indicates that even after only 3 weeks of the 60:20:20 diet, the RBG for females in Q5 was already greater than the other quintiles. This concurs with data from Experiment 1 with diet 73MBS (70:10:20) that relied on the 30-min OGTT after 2 weeks or 4 weeks on diet. While no significant differences were noted between other parameters across quintiles, this Experiment 4 stratification of females into RBG quintiles substantiates the *genetic permissiveness* that occurs early in this T2DM model, first noted in Experiment 1. Compared to human epidemiological data involving diet x genetic variation in T2DM, the Nile rat is more like humans and less like mice and conventional rats, as humans and Nile rats are CHO-sensitive while mice and rats are not [10,11,13,23,70,71].

## 4. Discussion

### 4.1. Early Age of Diabetes Onset

#### 4.1.1. Weanling rats

One significant observation from these experiments confirms and expands on our earlier implications that the Nile rat expresses detectable T2DM (i.e., elevated blood glucose) within weeks of weaning [12,14,59] when challenged with a hiCHO diet. By way of emphasis, impaired glucose metabolism, possibly implicating juvenile insulin resistance (IR) in childhood as well, initially appeared as a transient defect in weanling pups early during the first 4 weeks of a hiCHO diet challenge in Experiment 1, where females appeared slightly more impaired than males based on higher FBG, but not 30-min OGTT. This conclusion about transient IR derives from the observation that overnight food deprivation typically allowed an elevated RBG to reset to normal FBG (<60 mg/dl) by morning, i.e., the presumed IR, demonstrated previously linked to hyperinsulinemia and hyperleptinemia [12,60], appeared to dissipate if tissues in young Nile rats were allowed to experience a prolonged period of fasting, which is not unlike the diabetic prone Sand rat [72,73,74,75,76,77] and the recent emphasis on intermittent fasting as a prescribed method for arresting development of T2DM in humans [78]. 

A second aspect of this phenomenon surfaced when rats, which were eventually classified as ‘*resistant*’ to diabetes at the study’s end, were found retrospectively to have decreased their RBG during the course of the 10-week (Experiment 2) or 8-week (Experiment 3) diet challenges. The ‘corrective behavior’ was less robust when the GLoad was more severe in Experiment 3, indicating that ‘recovery’ likely depended on the stress induced by a continuous dietary Gload and whether or not sufficient time was allowed for tissues, especially muscle, liver and pancreatic beta cells, to recover from repeated meals providing the hiCHO challenge. The response in Experiments 2 and 3 was similar to results in Experiment 1, such that tissues recovered only in the slower growing, less voracious ‘*resistant’* pups. In other words, many rats initially seemed to ‘undergo metabolic adjustment’ early during the post-weaning challenge, improving with age if they voluntarily ate less food, i.e., reduced their Cumulative Gload, to eventually become ‘*resistant*’, while others failed to improve if they continued to eat excessively and ended up as ‘*susceptible*’. 

#### 4.1.2. FBG Less Sensitive Than RBG. OGTT More Sensitive

This combination of experiments lasting 2 to 10 weeks indicates that the RBG was more useful than FBG for predicting the onset and severity of diabetes in both male and female Nile rats, and that the higher GLoad in the 70:10:20 diet induced diabetes more rapidly and more severely in a shorter time than 60:20:20, which had double the percentage of fat calories. The RBG also predicted liver enlargement documented as steatosis of NAFLD previously [14,60] as a consequence of consuming hiCHO diets [79,80]. This was accompanied by an enlarged cecum, which presumably telegraphed changes in gut flora activity [81].

The ability of female rats in past experiments to mitigate their early diabetes as they matured, reducing RBG and OGTT as they approached sexual maturity (estrogen), contrasts with *susceptible* males (testosterone), which continued to develop diabetes based on their greater appetite and continued rapid growth [12,59]. The emphasis on male vulnerability to diabetes was further demonstrated by the progressively abnormal excursion in the OGTT that occurred between 6 weeks and 10 weeks, or 4 weeks and 8 weeks, of hiCHO diet challenges in *susceptible* rats (Experiments 2 and 3, respectively). These observations suggest that males, more than females, eventually lose their ability to cope metabolically with the high GLoad as they mature, if they consume too much CHO, gain weight too rapidly, and progressively lose beta-cell insulin production to become diabetic [14,35,59]. The sequence is exacerbated by consuming fiber-free hiCHO diets that overload tissues with rapidly absorbed glucose, or especially when sugar and fructose drive hepatic TG production and NAFLD [80,82,83] while inducing IR and hyperinsulinemia. Chronic insulinemia is itself a potent growth stimulus that enhances tissue growth and blood pressure elevation in prediabetes [84], a scenario that mirrors the T2DM and hypertension observed in these rats [59,60].

#### 4.1.3. Similarity to hiCHO in Childhood

Early post-weaning behavior relating eating patterns to CHO-driven food intake has been described as a potentially important aspect of early childhood obesity linked to implications for adverse cognitive development in children [85] and risk of developing obesity and T2DM [86,87]. In certain respects, accelerated early growth can be seen as an adaptive advantage to hasten sexual maturation to allow a species to maximize reproductive performance when the food supply is abundant. But an over-abundance of available energy as hiCHO has the potential of imprinting juvenile metabolism with the undesirable prospect of subsequently developing chronic disease in the form of T2DM [86,87]. In a similar manner, accelerated growth by premature infants fed formula diets are at a greater risk of developing MetS in early childhood than normal term infants fed breast milk [88,89]. By contrast, early childhood famine elicits the opposite effect, imprinting metabolism with a degree of protection against adult T2DM [90]. The link to changes in the microbiome based on response to the type of diet, especially hiCHO diet, that leads to early gut flora dysbiosis has been suggested [85,87,91]. An association between diet-altered gut flora and T2DM is well established in both animal models and humans [81,92,93] with the consequence that T2DM is a growing concern for young children consuming Western-type diets [86,87]. This scenario coupled with the current study, where cecum size increased in tandem with the diabetes, also serves to demonstrate how the Nile rat might function as a model for early nutritional imprinting to impact child development and global expression of a major chronic disease.

The present data further indicate the advantage of assessing the nutritional attributes of diabetes induction and progression in an appropriate animal model by beginning the dietary challenge early in life [85,87,88,89,91]. By weaning pups onto test diets at 3 weeks of age, these experiments on diabetes induction were limited to a more reasonable 8 to 10 week trial, and even 4 weeks, as in Experiment 1, all during the major growth period in Nile rats when metabolic imprinting by dietary factors is most apt to occur. In this fashion, and assuming one has the correct balance between *genetically permissive* pups with *‘resistant’* and *‘susceptible’* traits as outcomes, one might reasonably delineate the dietary factors involved in diabetes induction in the naïve metabolic system of the Nile rat, a metabolic circumstance apparently shared with another North African desert rodent, the Sand rat [72,73,74,75,76,77]. By contrast, most human epidemiologic studies of T2DM have been conducted in adults [10,11,36,37,38,39,40,41,42,43], which are arguably beyond the juvenile imprinting stage that favors induction and, thus, are less apt to detect the effects of dietary modulation on either MetS or diabetes.

### 4.2. Genetic Permissiveness

*Genetic permissiveness* is key to expression of *resistant* versus *susceptible* Nile rats. One persistent finding noted over the years [13,14,59], and referred to above, is the individual variation in response to dietary hiCHO challenge observed during T2DM induction in this model. Thus, the original description of ‘early onset’ or ‘late onset’ diabetes [59] is more effectively translated as rats *susceptible* or *resistant* to T2DM based on their RBG rising above 75 mg/dl, or not, when used to assess diet-induced diabetes progression. In the current report, this diabetes resistance-susceptibility was defined by the distribution of RBG across quintiles when Nile rats were challenged with a hiCHO diet [13,14]. This quintile stratification of RBG, or the 30-min OGTT, described relationships between diabetes and various physiological metagenetic parameters linked to the degree of diabetes expressed in these rats fed a single diabetogenic diet during each experiment. Stratification into quintiles not only disclosed the percentages of *resistant or susceptible* rats for a given diet challenge, but further revealed their associations between caloric intake, Cumulative GLoad, growth rate, and food efficiency, as well as metabolic parameters linked to the severity of diabetes recorded at necropsy.

In effect, the metagenetic parameters were linked most closely to diabetes depending on the GLoad of the hiCHO diet consumed, in this case as 70:10:20 in Experiments 1 and 3, or 60:20:20 diets in Experiment 2. All *susceptible* rats eventually develop diabetes by 6–8 months to the point where diet intervention would no longer have protected them against the disease progression, all having to do with age at intervention/induction and the stress inflicted by a given diet designed to cause hyperinsulinemia and eventually impair insulin production [13,14,35]. Hence, *genetic permissiveness* could be construed as susceptibility to inductive imprinting for MetS and T2DM by a diet at an early age that alters gut flora and microbiome metabolism [81,85,91,93]. It is unclear to what extent *resistance*/*susceptibility* is an inherent genetic trait, or reflects an epigenetic modification conferred by the postnatal environment [86], possibly including modification of gut immune cells via an altered microbiome [94] versus how much can be assigned specifically to the various diet challenges, particularly the GLoad and/ or content of fiber [14,40] or polyphenols [95] of the diet. This presumably represents a diet x gene interaction that varies according to the *genetic permissiveness* of each rat to respond to changes in the macronutrient composition that impacts the microbiome [13,86,93,94,96]. 

### 4.3. Diet CHO Quality and GLoad

It is important to note that these diets were all intentionally fiber-free to remove that ingredient as a variable in the T2DM outcome. As such, its absence rendered the diet more apt to increase the glycemic burden. Fiber is known to have anti-diabetic effects in Nile rats [14], as well as in humans, where it can reduce HbA1c [40,97,98].

This raises two points about the GLoad and diabetes risk. First, the Gload reflects the GI of the food source, in this case reflecting a mix of dextrose, sucrose or starch counterbalanced by the fat component [with its 0 Gload rating] in keeping with the calibrated system established for humans [10,40]. Varying the CHO type and amount caused the Gload to range between 224/2000 kcal (60:20:20 ratio, Experiments 2 & 4) and 259 or 295, both with a 70:10:20 diet energy ratio (Experiments 1 & 3). Second, rats in both Experiments 2 and 3 were exposed to similar nutritional paradigms based on hiCHO diets from 3 weeks of age and ate approximately the same number of calories for normal growth, despite the different sources of calories (CHO vs. fat), different dietary Gloads, and different caloric density of the diets. Those consuming the diet with more CHO and a greater GI/ Gload per kg diet (Experiment 3), ate 3–6 more calories per day (10–20%), even though that diet with less fat also contained less energy per gram. As a consequence, the diet in Experiment 3 (70:10:20), with 10% more energy from CHO and greater Gload, induced T2DM more rapidly and more severely than that in Experiment 2 (60:20:20), based on the RBG and the 30-min OGTT.

In other words, hiCHO calories in Experiment 3 were more devastating than calories derived from fat in Experiment 2 for diabetes induction. Thus, overall, the diet with a 295 Gload (70:10:20, Experiment 3) induced more diabetes during its 8 week trial, and the diabetes progressed from an earlier age than that with the 224 Gload (60:20:20 fed for 10 weeks in Experiment 2). The implication is that *susceptibility* to T2DM in these Nile rats may represent an inherent CNS behavioral issue linked to the genetics [or epigenetics] of failed satiety [excessive consumption] in the face of high CHO intake in the absence of fiber. The cecum weight also was increased in Q5 rats with severe diabetes, indicating that their distorted energy intake and altered metabolism associated with diabetes coincided with presumed changes in gut flora [81].

These data provide strong evidence that the GI and Gload are ultimately more important determinants of diabetes than the macronutrient composition per se [14], when all diet variables are carefully controlled between diets. Like these Nile rat studies, and consistent with the several unanswered questions about the importance macronutrient sources, in general, observational data from 85,000 nurses 30–55 years old revealed that their T2DM risk was directly related to the dietary GLoad, but a higher P/S ratio attributed to plant-based fat, as compared to fat in animal products, was associated with reduced diabetes risk, as well [41], leaving causal relationships in doubt. However, a recent meta-analysis involving more than 430,000 subjects and 25 years of follow-up to assess the impact of CHO intake on all-cause mortality, including complications from diabetes, revealed that macronutrient sources of protein and fat, especially from animal products, have a major detrimental effect on chronic disease risk that potentially rivals that from hiCHO-lowCHO-fiber relationships [43]. As with all previous epidemiological studies, it represented observational data in adults [10,11,39,40,41,42] and not the controlled dietary induction studies reported here in the weanling Nile rat model. 

Thus, it remains to be determined whether the modest improvement afforded by the lower CHO energy in Experiment 2 (60:20:20) was due to the removal of CHO or the increase in fat, i.e., was there something specific about the fat fed that was protective, or was it simply a reduction in the GLoad? Also, the protein source in these studies was animal in origin in the form of milk proteins, which may have enhanced the diabetes outcome [41,42,43]. Although the high correlation between Cumulative GLoad and diabetes induction in these rats favors the deleterious effect of the CHO load per se, more experiments are needed to rule out a fat or protein effect, even though the composition of the fat and protein in the two experiments was constant (fat being an American Fat Blend with a polyunsaturated/saturated fat (P/S) ratio of 0.33 for all diets).

### 4.4. Cumulative GLoad, Growth Rate and T2DM

Food intake and growth rate revealed significant parallels between the quintiles of RBG and metagenetic attributes describing the T2DM. These relationships, initially telegraphed by energy intake (kcal/day), were captured by the daily, and ultimately, by the Cumulative GLoad per rat, which became apparent during the first week of feeding. This differential in *genetic permissiveness* associated with hyperphagia (seemingly failed appetite control from the very beginning] across quintiles was maintained throughout the study and was mirrored in body weight gain. The consistency across Q1–Q4 for food efficiency suggests that food intake regulation, including the macronutrient composition, and the subsequent effect on growth rate might trigger an intrinsic signal that sets MetS and T2DM in motion. In a similar vein, accelerated growth rate in formula-fed premature infants has been associated with MetS in early childhood [88,89], and, as mentioned earlier, the initial diet history likely imprints energy metabolism via the gut flora and may include modified short chain fatty acid production by a microbiome lacking adequate fiber. For example, a role for enhanced appetite driven by acetic acid over-production has been described in a fat-fed mouse model [99]. Another intriguing possibility is the observation that an intestinal innate immune cell, i.e., CD11c dendritic cells, can modulate food intake and body mass gain in growing mice by signaling the hunger and satiety hypothalamic neurons. These neurons are also targets of energy-sensitive hormones, including insulin and leptin [94]. And CD11c activation requires cooperative action of commensal microbiota, which would depend on appropriate dietary CHO composition, including fiber, to main the integrity of the gut flora. Whether these concepts apply to Nile rats or premature human infants fed high GLoad diets is unknown. 

#### 4.4.1. Muscle Clock Gene

As noted above, the Cumulative GLoad appeared to be related to inherent body size more than to diet, which may have influenced hunger and increased food intake. Even energy utilization was possibly modulated by an intrinsic regulatory signal originating with the gut flora that only indirectly linked to an extrinsic factor in the diet. This apparent hyperphagia, starting with Q4, but mostly in Q5, continued throughout the diet trials until a ravenous appetite and thirst were encountered at the end of study in the most *susceptible* rats showing clinical signs of ketosis (Q5). The food intake data are further reflected in body weight gain per day with the most *resistant* rats (Q1), and to lesser extent with Q2 and Q3, revealing the lowest intakes and least weight gain. Thus, it is not clear whether food intake was driven genetically by poor satiety feedback signals or possibly by gene-controlled growth and an expanding muscle mass, e.g., possibly involving the ‘muscle clock effect’, triggering an increased appetite for more food [100] or the afore-mentioned CD11c cell activation by appropriate gut bacteria [94]. However, unlike fat-fed mouse and rat models of obesity-driven T2DM, where body fat often exceeds 50% of daily weight gain, these Nile rats never developed obesity, as the 3 major fat depots combined never exceed 15% of the total body weight [59], even though expanding body fat tended to track weight gain in all tissues in concert with the excess calories consumed.

In any event, a striking aspect of the data set in Experiments 2 and 3 was the link between caloric intake, growth rate, and diabetes severity (RBG and 30-min OGTT). The implication is that excess energy intake (kcal/day, including the GLoad) was critical to diabetes onset and outcome, as it directly impacted growth rate affecting tissue expansion and IR. Note that linear growth [bone and skeletal growth] as expressed in body length tended not to be affected, only organ expansion of soft tissues seemed to be altered. One possibility is that IR is a response to limitations in adipose, liver, and muscle expansion relative to the mass of glucose consumed and disposed of per unit time postprandially. Again, this might impact the aforementioned ‘muscle clock gene’ *Bamal1*, which is controlled in turn by muscle circadian rhythm to regulate glucose homeostasis in muscle and liver [101] or the CD11c cell influence on food intake and body mass [94].

#### 4.4.2. Bile Acids

In a similar vein it is known that bile acids interact with hormone farnesoid X receptor (FXR) and the membrane G protein-coupled bile acid receptor (TGR5), which modulate secretion of incretin hormones and fibroblast growth factor 19 (FGF19) by intestinal L-cells. The latter endocrine hormone controls systemic energy expenditure and cholesterol metabolism in the liver, as well [102]. In healthy individuals, bile acid metabolism is regulated by circadian cycles of fasting and refeeding via feedback from a healthy microbiome. A hiCHO diet that promotes hyperphagia and reduces fasting times, would predictably exert a detrimental effect on the gut flora [80], disrupting the profile and production of primary and secondary bile acids to adversely impact FXR and regulation of glucose and lipid metabolism [102]. Overproduction of secondary bile acids by an unhealthy gut flora is also associated with liver tumors [103], which are frequently encountered in retired breeder Nile rats older than 1 year with advanced T2DM [13]. Furthermore, the gut flora regulates bile acid metabolism [104], and bile acids themselves are known to influence gut flora activity [105], so the relationship is reciprocal, with diet composition being the primary factor affecting both. Further studies of these complex relationships to diet and diabetes in the Nile rat are warranted.

## 5. Conclusions

These observations appear to be novel because we can find no evidence of CHO-induced T2DM in any animal, including humans, that demonstrates diabetes outcome is related directly to the Cumulative GLoad assessed throughout the experiment (not practical for long-term clinical studies or observational studies). Nonetheless, T2DM risk related to the GI and increasing GLoad has been inferred on a number of occasions based on indirect observational data, in general [10,11], or in women [41], but not men [42]. Thus, these data have resulted in somewhat inconsistent conclusions and lingering questions about GI and GLoad as risk factors for human T2DM [38,40] and only now are more substantial evidence-supported conclusions being drawn about the detrimental impact of long-term histories of dietary GI and GLoad [10,11,43]. 

The Nile rat appears uniquely suited to modelling human metabolic sensitivity to dietary CHO quality under properly controlled dietary conditions. This conclusion has important public health implications, now that proof of concept has been modelled by these experiments. One implication is that observational nutritional data in adults, even when enrolled as nondiabetic, can only weakly predict long term diabetes outcome attributed to diet unless or until the Cumulative GLoad can be better assessed, possibly for a number of years, and/or that the T2DM risk conferred by diet more appropriately reflects the diet-based induction of T2DM at an early age [85,86,87,90,91]. Our data on Nile rat variation related to *genetic permissiveness* appear similar to those in human populations, indicating that a large number of rats per diet (*n =* 10–15) is required in such preclinical experiments to detect/illustrate these diet x gene interactions in this model. Our earlier observations, some based on longer term studies, described rats as ‘early responders’ versus ‘late responders’ in terms of their diabetes induction during the course of prolonged experiments of several months duration [59].

In conclusion, using quintiles of RBG and 30-min OGTT to stratify large cohorts of Nile rats fed hiCHO challenge diets (representing 3 GLoads) in four experiments proved to be an effective way to assess the diet x gene interactions and express the host *genetic permissiveness* that dictates susceptibility to induction and progression of T2DM in the model. Excessive caloric intake associated with a high GLoad immediately after weaning was a clear predictor of their final quintile distribution for diabetes outcome. Thus, the strong *genetic permissiveness* aspect of the onset and progression of the disease, possibly involving epigenetic changes operating via the gut flora, underscores the robustness of the Nile rat nutritional model for study of T2DM in humans.

## Figures and Tables

**Table 1:** Diet Compositions.

Brandeis Diet #	Diet 133	Diet 73MBS	Diet 73MB
Experiment #	2,4	1,3	3
CHO:Fat:Protein %Energy	60:20:20	70:10:20	70:10:20
(Fat/Protein % Energy ratio)	1.0	0.5	0.5
kcal/g	4.22	3.98	3.98
GLoad per 2000 kcal	224	259	295
**Ingredients**	**g/kg diet**		
Casein	106	100	100
Lactalbumin	106	100	100
Dextrose	186	200	350
Sucrose	186	200	0
Cornstarch *	200 (+60 gel)	238 (+60 gel)	288 (+60 gel)
Fat [average American Fat Blend]			
Margarine B (94% fat w butter) **	100 (94 as fat)	47 (44 as fat)	47 (44 as fat)
butter (fat component only)	21	6	6
tallow	46	15	15
lard	15	0	0
soybean oil	18	23	23
SFA:MUFA:PUFA ratio ^##^	45:40:15	45:40:15	45:40:15
P/S ratio ^#^	0.33	0.33	0.33
Mineral mix, Ausman-Hayes ^a^	46	44	44
Vitamin mix, Hayes-Cathcart ^b^	12	11	11
Choline chloride (75% choline)	3	3	3
Cholesterol (0.06%)	0.6	0.6	0.6

* 60 g cornstarch added to 800 mL hot water for gel prior to mixing in the ingredients. ** Margarine B: Average American Fat Blend. - butter, tallow, lard, soybean oil as (21:46:15:18 weight% ratio). ^#^ P/S—Polyunsaturated Fat/Saturated Fat. ^##^ SFA—Saturated Fatty acid; MUFA—Monounsaturated Fatty acid; PUFA—Polyunsaturated fatty acid. ^a,b^ Leow et al. (2016) [68].

**Table 2:** Study Designs for Experiments 1–4 set to examine Genetic Permissiveness by using final quintiles of Blood Glucose. All Nile Rats were weaned at 3 weeks of age and studies varied from 2–10 weeks.

Experiment	Table	Diet (CHO:Fat:Protein %Energy Ratio)	Length of Study (Weeks)	Sex of Nile Rat	Energy Intake (Week on Study)		RBG (Week on Study)	FBG (Week on Study)	OGTT	OGTT	Necropsy (Weeks on Study)	
					Kcal/day (Weeks on Study)	Body Weight Gain/Day (Weeks on Study)			30 min (Week on Study)	60 min (Week on Study)	Organ Weights	Plasma Lipids
1	3	73MBS (70:10:20)	2,4	M	na	na	na	✓ (2,4)	✓ (2,4)	na	na	na
		73MBS (70:10:20)	2,4	F	na	na	na	✓ (2,4)	✓ (2,4)	na	na	na
2	4	133 (60:20:20)	10	M	✓ (1–9)	✓ (1–9)	✓ (6,10)	✓ (6,10)	✓ (6,10)	✓ (6,10)	✓ (10)	✓ (10)
3	5	73MB/73MBS (70:10:20)	8	M	✓ (1–7)	✓ (1–7)	✓ (4,8)	✓ (4,8)	✓ (4,8)	✓ (4,8)	✓ (8)	✓ (8)
4	6	133 (60:20:20)	3	F	na	na	✓ (3)	✓ (3)	✓ (3)	✓ (3)	✓ (3)	na

**Table 3:** **(Experiment 1)**. (**a**) Quintiles based on the 30’-OGTT for 255 3-week old male Nile rats after being housed with littermates immediately upon weaning for either 2 or 4 weeks and fed a hiCHO diet (Diet 73MBS, NR Study 129); (**b**) Quintiles based on the 30’-OGTT for 247 3-week old female Nile rats after being housed with littermates immediately upon weaning for either 2 or 4 weeks and fed a hiCHO diet (Diet 73MBS, NR Study 129).

**CHO:Fat:Protein%energy**	Diet 73MBS (70:10:20) *					Diet 73MBS (70:10:20) *				
**(a) Males**										
	**Housed together for 2 weeks (*n =* 165)**					**Housed together for 4 weeks (*n =* 90)**				
**Quintile**	**1**	**2**	**3**	**4**	**5**	**1**	**2**	**3**	**4**	**5**
**30’-OGTT range (mg/dl)**	(59–61)	(59–161)	(214–241)	(245–292)	(296–435)	(44–98)	(103–149)	(150–192)	(193–257)	(260–525)
**Fasting Body Weight (g)**	56 ± 9^abc^	57 ± 7	60 ± 10^a^	61 ± 7^b^	61 ± 7^c^	67 ± 11	65 ± 7^ab^	67 ± 7	71 ± 10^a^	72 ± 9^b^
	**Oral Glucose Tolerance Test (mg/dl) after 2 weeks**					**Oral Glucose Tolerance Test (mg/dl) after 4 weeks**				
FBG, 0 min	55 ± 20^a^	54 ± 19^b^	55 ± 16^c^	62 ± 23	65 ± 21^abc^	41 ± 10^ab^	51 ± 27^c^	47 ± 11^d^	59 ± 23^a^	67 ± 34^bcd^
30 min	121 ± 28^abcd^	193 ± 15^aefg^	227 ± 9^behi^	270 ± 14^cfhj^	337 ± 37^dgij^	82 ± 15^abcd^	127 ± 15^aefg^	174 ± 13^behi^	236 ± 17^cfhj^	336 ± 79^dgij^
**(b) Females**										
	**Housed together for 2 weeks (*n =* 138)**					**Housed together for 4 weeks (*n =* 109)**				
**Quintile**	**1**	**2**	**3**	**4**	**5**	**1**	**2**	**3**	**4**	**5**
**30’-OGTT range (mg/dl)**	(86–138)	(140–175)	(178–216)	(216–270)	(272–427)	(40–105)	(106–139)	(141–174)	(175–216)	(223–398)
**Fasting Body Weight (g)**	44 ± 7^ab^	47 ± 7	46 ± 6^c^	51 ± 8^ac^	49 ± 8^b^	47 ± 9^a^	48 ± 10^b^	51 ± 12	55 ± 11^ab^	51 ± 10
	**Oral Glucose Tolerance Test (mg/dl) after 2 weeks**					**Oral Glucose Tolerance Test (mg/dl) after 4 weeks**				
FBG, 0 min	56 ± 18^a^	58 ± 17^b^	61 ± 18^c^	68 ± 20	74 ± 34^abc^	52 ± 16^ab^	60 ± 21	68 ± 40^a^	64 ± 15	76 ± 27^b^
30 min	111 ± 14^abcd^	159 ± 11^aefg^	197 ± 11^behi^	246 ± 18^cfhj^	318 ± 47^dhij^	82 ± 18^abcd^	124 ± 9^aefg^	156 ± 11^behi^	196 ± 15^cfhj^	279 ± 43^dgij^

* Represents Diet 73MBS, CHO:Fat:Protein %energy with GLoad of 259/2000 kcal; 4.0 kcal/g dry diet.

**Table 4:** **(Experiment 2).** Quintiles of RBG along with diabetes metagenetic profile for 90 male Nile rats (3 weeks old) fed semipurified hiCHO Diet 133 (60:20:20)* for 10 weeks, then subdivided into ’resistant’ or ’susceptible’ quintiles based on RBG <75 mg/dl>, respectively. (NR Studies 130, 132, 144, 148, 150, 151, 156).

**Diet (CHO:Fat:Protein%energy)**	**Diet 133** (60:20:20) *				
**Quintiles by RBG**	**Q1**	**Q2**	**Q3**	**Q4**	**Q5**
**T2DM ’genetic permissiveness’ ranked by quintiles**	**Resistant**	**Resistant**	**Susceptible–preDiabetic**	**Susceptible–moderate**	**Susceptible–severe**
***n =***	**18**	**18**	**18**	**18**	**18**
**RBG (range) after 10 weeks on diet (mg/dl)**	**(43–58)**	**(58–66)**	**(66–82)**	**(82–237)**	**(270–600)**
after 6 weeks diet	66 ± 16^a^	73 ± 19^b^	70 ± 19^c^	68 ± 18^d^	295 ± 179^abcd^
after 10 weeks diet	53 ± 4^ab^	62 ± 3^cd^	72 ± 5^ef^	158 ± 54^aceg^	439 ± 119^bdfg^
	**Body Weight (g)**				
Initial (3 weeks old)	31 ± 5	30 ± 4	32 ± 3	31 ± 4	32 ± 5
after 6 weeks diet	82 ± 9^abcd^	88 ± 5^a^	87 ± 7^b^	90 ± 6^c^	92 ± 8^d^
after 10 weeks diet	94 ± 10^abcd^	101 ± 8^ae^	103 ± 8^b^	107 ± 6^cef^	100 ± 11^df^
	**Body Weight Gain per day (g/day for 9 weeks)**				
1–6 week average	1.18 ± 0.22^abc^	1.34 ± 0.19^a^	1.26 ± 0.15^d^	1.37 ± 0.15^b^	1.39 ± 0.19^cd^
7–10 week average	0.46 ± 0.25^a^	0.45 ± 0.23^b^	0.54 ± 0.12^cd^	0.72 ± 0.49^abce^	0.31 ± 0.17^de^
Total average (after 9 weeks)	1.00 ± 0.22^a^	1.09 ± 0.18^b^	1.09 ± 0.11^c^	1.22 ± 0.18^abcd^	1.06 ± 0.19^d^
**Water Intake per day in the** **9th week (ml/day)**	5 ± 2^a^	4 ± 1^b^	4 ± 1^c^	5 ± 2^d^	22 ± 27^abcd^
	**Energy Intake (kcal/day)**				
1^st^ week	32 ± 4^ab^	33 ± 3	33 ± 3^c^	34 ± 3^a^	35 ± 4^bc^
2^nd^ week	32 ± 3^abc^	34 ± 3^ad^	34 ± 3^e^	36 ± 2^b^	37 ± 4^cde^
6^th^ week	29 ± 5^ab^	31 ± 4^c^	31 ± 3^d^	34 ± 2^ae^	39 ± 10^bcde^
8^th^ week	31 ± 6^ab^	35 ± 5^c^	34 ± 4^d^	37 ± 3^ae^	45 ± 15^bcde^
9^th^ week	32 ± 5^ab^	35 ± 6^c^	33 ± 5^d^	38 ± 4^ae^	46 ± 13^bcde^
Total average (after 9 weeks)	31 ± 3^ab^	33 ± 3^c^	33 ± 2^d^	35 ± 1^ae^	40 ± 8^bcde^
	**Glycemic Load**				
per kg body weight at 10 weeks	37 ± 4^a^	37 ± 3^b^	36 ± 2^c^	37 ± 3^d^	46 ± 15^abcd^
per rat/day	3.48 ± 0.30^ab^	3.74 ± 0.29^c^	3.70 ± 0.25^d^	3.97 ± 0.15^ae^	4.46 ± 0.92^bcde^
per BMI/rat/day	0.66 ± 0.07^a^	0.70 ± 0.06^b^	0.67 ± 0.04^c^	0.71 ± 0.04^d^	0.88 ± 0.32^abcd^
Cumulative GLoad /rat for 10 weeks	243 ± 21^ab^	262 ± 20^c^	259 ± 17^d^	278 ± 11^ae^	312 ± 64^bcde^
	**Food Efficiency (g gained /1000 kcal)**				
1–6 week Average	38 ± 6	41 ± 5	39 ± 4	40 ± 4	38 ± 8
7–10 week Average	15 ± 7^ab^	13 ± 7^cd^	16 ± 3^e^	20 ± 14^acf^	8 ± 5^bdef^
Total average (after 9 weeks)	32 ± 6^a^	33 ± 5^b^	33 ± 3^c^	35 ± 6^d^	28 ± 7^abcd^
	**Oral Glucose Tolerance Test (mg/dl) after 6 weeks**				
FBG, 0 min	44 ± 10^ab^	49 ± 9^c^	51 ± 9^d^	57 ± 12^a^	62 ± 23^bcd^
30 min	147 ± 39^abc^	181 ± 58^de^	214 ± 62^af^	242 ± 67^bdg^	366 ± 91^cefg^
	**Oral Glucose Tolerance Test (mg/dl) after 10 weeks**				
FBG, 0 min	49 ± 14^a^	49 ± 9^b^	45 ± 10^c^	53 ± 14^d^	76 ± 47^abcd^
30 min	147 ± 47^abc^	171 ± 49^de^	221 ± 46^af^	264 ± 114^bdg^	432 ± 123^cefg^
60 min	113 ± 56^ab^	103 ± 62^cd^	136 ± 48^e^	173 ± 93^acf^	331 ± 143^bdef^
	**Organ weight (%BW)**				
Liver	3.56 ± 0.47^a^	3.49 ± 0.50^b^	3.50 ± 0.24^c^	3.60 ± 0.33^d^	5.16 ± 1.23^abcd^
Kidney	0.70 ± 0.06^a^	0.69 ± 0.06^b^	0.68 ± 0.04^c^	0.73 ± 0.08^d^	1.02 ± 0.28^abcd^
Cecum	1.17 ± 0.17^a^	1.08 ± 0.33^b^	1.12 ± 0.18^c^	1.00 ± 0.15^d^	1.57 ± 0.69^abcd^
	**Adipose (%BW)**				
Epididymal	2.85 ± 0.67^a^	2.96 ± 0.69^b^	2.90 ± 0.58^c^	3.02 ± 0.56^d^	2.24 ± 0.70^abcd^
Perirenal	1.36 ± 0.47^ab^	1.72 ± 0.48^c^	1.95 ± 0.53^ad^	1.97 ± 0.37^be^	1.33 ± 0.85^cde^
Brown fat	1.77 ± 0.44^ab^	2.09 ± 0.48^c^	2.37 ± 0.52^ad^	2.59 ± 0.41^bce^	1.73 ± 0.78^de^
Total fat	5.97 ± 1.31^ab^	6.57 ± 1.37^c^	7.03 ± 1.16^ad^	7.18 ± 1.29^be^	5.16 ± 2.12^cde^
**Carcass (%BW)**	75 ± 2^ab^	74 ± 2^c^	74 ± 1^d^	73 ± 2^a^	72 ± 2^bcd^
**Length (cm)**	13.2 ± 0.4^abc^	13.5 ± 0.5^a^	13.3 ± 0.4	13.6 ± 0.3^b^	13.6 ± 0.6^c^
**Body Mass Index (kg/m^2^) at 10 weeks**	5.27 ± 0.48^a^	5.40 ± 0.52	5.57 ± 0.34^b^	5.65 ± 0.26^ac^	5.23 ± 0.66^bc^
	**Plasma (mg/dl)**				
Total Cholesterol	123 ± 23^a^	113 ± 26^b^	116 ± 13^c^	131 ± 39^d^	322 ± 410^abcd^
Total Triglycerides	70 ± 26^a^	74 ± 25^b^	68 ± 25^c^	96 ± 20^d^	213 ± 298^abcd^

* Represents CHO:Fat:Protein % energy with GLoad of 224/2000kcal; 4.2 kcal/g dry diet. Values are mean ± SD. ^a, b^ Means in a row sharing common superscripts are significantly different (*P* < 0.05) by one-way ANOVA and Fisher’s PLSD test. ^#^ *n =* 18 per Quintile, total 90 rats.

**Table 5:** **(Experiment 3).** Quintiles of RBG along with diabetes metagenetic profile for 32 male Nile Rats (3 weeks old) fed semipurified high CHO Diet 73 (70:10:20)* for 8 weeks, then subdivided into ’resistant’ or ’susceptible’ quintiles based on RBG <75 mg/dl>, respectively (NR Studies 127, 128, 137).

**Diet** **(CHO:Fat:Protein %energy)**	**Diet 73** (70:10:20) *				
**Quintiles by RBG**	**Q1**	**Q2**	**Q3**	**Q4**	**Q5**
**T2DM ’genetic permissiveness’ ranked by quintiles**	**Resistant**	**Resistant**	**Susceptible**	**Susceptible**	**Susceptible**
***n =***	**6**	**6**	**6**	**7**	**7**
**RBG (range) after 8 weeks on diet (mg/dl)**	**(43–63)**	**(65–86)**	**(88–286)**	**(294–444)**	**(469–600)**
after 4 weeks on diet	65 ± 9^ab^	68 ± 6^cd^	107 ± 33^ef^	217 ± 111^ace^	283 ± 128^bdf^
after 8 weeks on diet	53 ± 7^abc^	78 ± 10^def^	185 ± 85^adgh^	369 ± 50^begi^	545 ± 58^cfhi^
	**Body Weight (g)**				
Initial (3 weeks of age)	36 ± 6	35 ± 10	32 ± 6	35 ± 8	36 ± 9
after 4 weeks on diet	76 ± 5^a^	74 ± 6^b^	78 ± 6	85 ± 8^abc^	77 ± 7^c^
estimated at 6 weeks on diet	85 ± 4	83 ± 7^a^	88 ± 5	92 ± 8^ab^	83 ± 4^b^
after 8 weeks on diet	93 ± 5	92 ± 7^a^	97 ± 6	99 ± 9^b^	89 ± 6^ab^
	**Body Weight Gain per day (g/day for 7 weeks)**				
1–4 week average	1.64 ± 0.66	1.32 ± 0.16^a^	2.02 ± 1.03^ab^	1.84 ± 0.23	1.38 ± 0.20^b^
5–7 week average	0.69 ± 0.22^a^	0.70 ± 0.12^b^	0.62 ± 0.21	0.53 ± 0.13	0.38 ± 0.38^ab^
Total average (after 7 weeks)	1.26 ± 0.11^a^	1.15 ± 0.16^b^	1.39 ± 0.33^bc^	1.34 ± 0.11^d^	1.01 ± 0.22^acd^
	**Energy Intake (kcal/day)**				
1st week	34 ± 3	32 ± 3	33 ± 3	35 ± 4	36 ± 6
2nd week	34 ± 2	33 ± 3	36 ± 2	37 ± 4	37 ± 6
3rd week	34 ± 4^a^	33 ± 2^b^	36 ± 3	39 ± 3^ab^	37 ± 6
4th week	31 ± 3^a^	30 ± 4^bc^	33 ± 2	36 ± 4^ab^	35 ± 5^c^
5th week	34 ± 2^ab^	34 ± 4^cd^	37 ± 4	40 ± 5^ac^	42 ± 6^bd^
6th week	34 ± 2^a^	34 ± 4^b^	37 ± 4^c^	40 ± 4^d^	46 ± 10^abcd^
7th week	33 ± 2^ab^	35 ± 4^cd^	37 ± 3^e^	41 ± 4^acf^	50 ± 10^bdef^
Total average (After 7 weeks)	35 ± 1^a^	34 ± 3^bc^	37 ± 3	40 ± 3^ab^	39 ± 6^c^
	**Glycemic Load**				
per kg body weight at 8 weeks	54 ± 2^a^	52 ± 3^b^	56 ± 3^c^	59 ± 6^d^	67 ± 11^abcd^
per rat/day	5.03 ± 0.22^ab^	4.81 ± 0.46^cd^	5.42 ± 0.41	5.81 ± 0.46^ac^	5.96 ± 0.96^bd^
per BMI/rat/day	1.01 ± 0.56^ab^	0.97 ± 0.09^cd^	1.08 ± 0.12^e^	1.19 ± 0.07^ac^	1.26 ± 0.23^bde^
Cumulative GLoad/rat for 8 weeks	282 ± 12^ab^	269 ± 26^cd^	304 ± 23	326 ± 26^ac^	334 ± 54^bd^
	**Food Efficiency (g gained /1000 kcal)**				
1–4 week average	48 ± 15	42 ± 7	57 ± 26^a^	50 ± 7	39 ± 7^a^
estimated 1–6 week average	37 ± 5^a^	37 ± 6	40 ± 7^b^	37 ± 3^c^	30 ± 8^abc^
5–7 week average	21 ± 7^a^	22 ± 5^bc^	17 ± 6	14 ± 3^b^	10 ± 12^c^
Total average (After 7 weeks)	37 ± 2^a^	36 ± 6^b^	38 ± 7^c^	34 ± 3^d^	26 ± 10^abcd^
	**Oral Glucose Tolerance Test (mg/dl) at 4 weeks**				
FBG, 0 min	51 ± 11	47 ± 8^a^	61 ± 12^abc^	43 ± 7^b^	48 ± 14^c^
30 min	164 ± 40^abc^	182 ± 68^de^	240 ± 74^a^	258 ± 71^bd^	269 ± 57^ce^
	**Oral Glucose Tolerance Test (mg/dl) at 8 weeks**				
FBG, 0 min	43 ± 5^a^	55 ± 16	50 ± 12^b^	56 ± 27	90 ± 64^ab^
30 min	162 ± 64^abc^	199 ± 40^de^	265 ± 72^afg^	364 ± 95^bdf^	405 ± 86^ceg^
	**Organ weight (%BW)**				
Liver	3.24 ± 0.23^abc^	3.56 ± 0.27^de^	3.85 ± 0.43^af^	4.34 ± 0.43^bd^	4.67 ± 0.84^cef^
Kidney	0.71 ± 0.03^a^	0.70 ± 0.06^bc^	0.75 ± 0.06^de^	0.92 ± 0.15^bdf^	1.07 ± 0.11^acef^
Cecum	1.17 ± 0.24^a^	1.15 ± 0.20^b^	1.03 ± 0.19^c^	1.39 ± 0.31^d^	2.20 ± 0.91^abcd^
	**Adipose (%BW)**				
Epididymal	3.29 ± 0.74^a^	3.19 ± 0.80^b^	3.01 ± 0.49^c^	3.12 ± 0.50^d^	2.10 ± 0.53^abcd^
Perirenal	1.59 ± 0.35^a^	1.38 ± 0.34^b^	1.72 ± 0.53^c^	1.28 ± 0.20^d^	0.72 ± 0.72^abcd^
Brown fat	1.86 ± 0.37^a^	2.28 ± 0.24^b^	2.27 ± 0.44^c^	1.97 ± 0.28^d^	1.19 ± 0.53^abcd^
Total fat	4.21 ± 2.74	5.26 ± 2.59	5.55 ± 2.88	4.42 ± 2.09	3.33 ± 2.18
**Carcass (%BW)**	72 ± 3	73 ± 2	73 ± 1	73 ± 1	73 ± 1
**Length (cm)**	13.6 ± 0.6	13.5 ± 0.4^a^	13.7 ± 0.6	14.1 ± 0.4^ab^	13.3 ± 0.5^b^
**Body Mass Index (kg/m^2^) based on 8 week FBW**	4.97 ± 0.39	4.97 ± 0.54	5.05 ± 0.37	4.90 ± 0.35	4.77 ± 0.53
	**Plasma (mg/dl)**				
Total Cholesterol	124 ± 25^a^	119 ± 17^b^	75 ± 12^c^	117 ± 42^d^	250 ± 83^abcd^
Total Triglycerides	65 ± 9^a^	93 ± 15	105 ± 46	127 ± 24^a^	88 ± 30

* Represents CHO:Fat:Protein %energy with GLoad of 295/2000kcal; 4.0 kcal/g dry diet. Values are mean ± SD. ^a, b^ Means in a row sharing common superscripts are significantly different (*P* < 0.05) by one-way ANOVA and Fisher’s PLSD test. ^#^ *n =* 6–7 per Quintile, total 32 rats.

**Table 6:** **(Experiment 4).** Quintiles of RBG along with diabetes metagenetic profile for 30 female Nile Rats (3 weeks old) fed semipurified hiCHO Diet 133 (60:20:20)* for 3 weeks, then subdivided into ’resistant’ or ’susceptible’ quintiles based on RBG <75 mg/dl>, respectively (NR Studies 133, 133(2), 139).

**Diet** **(CHO:Fat:Protein %energy)**	**Diet 133** (60:20:20) *				
**Quintiles by RBG**	**Q1**	**Q2**	**Q3**	**Q4**	**Q5**
**T2DM ’genetic permissiveness’ ranked by quintiles**	**Resistant**	**Resistant**	**Susceptible**	**Susceptible**	**Susceptible**
***n =***	**6**	**6**	**6**	**6**	**6**
**RBG (range) after 3 weeks (mg/dl)**	**(51–64)**	**(65–74)**	**(74–80)**	**(80–88)**	**(89–215)**
Ave after 3 weeks	60 ± 5^a^	69 ± 4^b^	77 ± 3^c^	82 ± 3^d^	123 ± 49^abcd^
	**Body Weight (g)**				
Initial (3 weeks of age)	27 ± 3	30 ± 6	30 ± 4	28 ± 4	28 ± 2
after 3 weeks on diet	57 ± 9	57 ± 5	61 ± 4	59 ± 4	56 ± 10
	**Oral Glucose Tolerance Test (mg/dl) after 3 weeks**				
FBG, 0 min	64 ± 25	51 ± 11	51 ± 11	69 ± 25	70 ± 18
30 min	159 ± 48^a^	193 ± 67	243 ± 56^a^	168 ± 41	225 ± 109
	**Organ weight (%BW)**				
Liver	3.55 ± 0.52	3.37 ± 0.34	3.88 ± 0.6^a^	3.23 ± 0.29^ab^	3.93 ± 0.56^b^
Kidney	0.7 ± 0.09	0.73 ± 0.06	0.67 ± 0.06	0.66 ± 0.05	0.67 ± 0.09
Cecum	1.42 ± 0.16	1.61 ± 0.53	1.44 ± 0.26	1.36 ± 0.36	1.56 ± 0.4
	**Adipose**				
Epididymal	1.97 ± 0.56	2.16 ± 0.84	2.33 ± 0.48	2.54 ± 0.49	2.06 ± 0.6
Perirenal	1.16 ± 0.6	0.98 ± 0.27^a^	1.26 ± 0.47	1.5 ± 0.46^a^	1.25 ± 0.27
Brown fat	2.07 ± 0.58	2.16 ± 0.18	2.47 ± 0.45	2.42 ± 0.27	2.24 ± 0.62
Total fat	4.34 ± 1.64	4.65 ± 2.04	4.73 ± 2.04	5.65 ± 1.66	4.65 ± 1.72
**Carcass (%BW)**	73 ± 10	77 ± 1	76 ± 1	76 ± 1	77 ± 2
**Length (cm)**	11.4 ± 0.4	11.5 ± 0.5	11.9 ± 0.2	11.6 ± 0.4	11.5 ± 0.7
**Body Mass Index (kg/m^2^) based on 3 week FBW**	4.42 ± 0.46	4.32 ± 0.34	4.31 ± 0.27	4.38 ± 0.52	4.24 ± 0.54

* Represents CHO:Fat:Protein % energy with GLoad 224/2000kcal; 4.2 kcal/g dry diet. Values are mean ± SD. ^a, b^ Means in a row sharing common superscripts are significantly different (*P* < 0.05) by one-way ANOVA and Fisher’s PLSD test. ^#^ *n =* 6 per Quintile, total 30 rats.
